# Neuroprotective Effects of Functionalized Hydrophilic Carbon Clusters: Targeted Therapy of Traumatic Brain Injury in an Open Blast Rat Model

**DOI:** 10.3390/biomedicines12122832

**Published:** 2024-12-13

**Authors:** Parasuraman Padmanabhan, Jia Lu, Kian Chye Ng, Dinesh Kumar Srinivasan, Kumar Sundramurthy, Lizanne Greer Nilewski, William K. A. Sikkema, James M. Tour, Thomas A. Kent, Balázs Gulyás, Jan Carlstedt-Duke

**Affiliations:** 1 Lee Kong Chian School of Medicine, Nanyang Technological University, Singapore 636921, Singapore; ljia@dso.org.sg (J.L.); kumarsundramurthy@hotmail.com (K.S.); 2 Defence Science Organisation National Laboratories, Singapore 117510, Singapore; nkianchy@dso.org.sg; 3 Department of Anatomy, Yong Loo Lin School of Medicine, National University Singapore, Singapore 119228, Singapore; dineshkumar@nus.edu.sg; 4 Department of Chemistry, Rice University, Houston, TX 77005, USA; lizanne.nilewski@gmail.com (L.G.N.); william.sikkema@gmail.com (W.K.A.S.); tour@rice.edu (J.M.T.); tkent@tamu.edu (T.A.K.); 5 Institute of Biosciences and Technology, Texas A&M University, Houston, TX 77030, USA; 6 President’s Office (Retired), Nanyang Technological University, Singapore 639798, Singapore

**Keywords:** traumatic brain injury (TBI), poly-ethylene-glycol-functionalized hydrophilic carbon clusters (PEG-HCCs), biologically compatible carbon-based nanoclusters, open blast rat TBI model

## Abstract

Traumatic brain injury (TBI) causes multiple cerebrovascular disruptions and oxidative stress. These pathological mechanisms are often accompanied by serious impairment of cerebral blood flow autoregulation and neuronal and glial degeneration. Background/Objectives: Multiple biochemical cascades are triggered by brain damage, resulting in reactive oxygen species production alongside blood loss and hypoxia. However, most currently available early antioxidant therapies lack capacity and hence sufficient efficacy against TBI. The aim of this study was to test a novel catalytic antioxidant nanoparticle to alleviate the damage occurring in blast TBI. Methods: TBI was elicited in an open blast rat model, in which the rats were exposed to the effects of an explosive blast. Key events of the post-traumatic chain in the brain parenchyma were studied using immunohistochemistry. The application of a newly developed biologically compatible catalytic superoxide dismutase mimetic carbon-based nanocluster, a poly-ethylene-glycol-functionalized hydrophilic carbon cluster (PEG-HCC), was tested post-blast to modulate the components of the TBI process. Results: The PEG-HCC was shown to significantly ameliorate neuronal loss in the brain cortex, the dentate gyrus, and hippocampus when administered shortly after the blast. There was also a significant increase in endothelial activity to repair blood–brain barrier damage as well as the modulation of microglial and astrocyte activity and an increase in inducible NO synthase in the cortex. Conclusions: We have demonstrated qualitatively and quantitatively that the previously demonstrated antioxidant properties of PEG-HCCs have a neuroprotective effect after traumatic brain injury following an explosive blast, acting at multiple levels of the pathological chain of events elicited by TBI.

## 1. Introduction

Traumatic brain injury (TBI) is a result of a physical assault to the brain and is a major cause of death and disability worldwide, especially in developed countries. In addition, the prevalence of TBI has risen dramatically in recent years due to terrorism, wars, and armed conflicts worldwide. It has been estimated that TBI results in a global economic burden of approximately USD 400 billion yearly spent on TBI-related medical care, hospitalization, and rehabilitation [1]. The frequency of TBI is highest among children and young adults due to accidents, violence, and sports, and it is more frequent in males than females [2]. Between 2007 and 2015, the Veterans Health Administration screened one million combat veterans for TBI and 8.4% of them were diagnosed with TBI [3]. The global prevalence of blast injuries has increased between 2007 and 2017 with more than a tripling of terrorist attacks, and the US Department of Defense described blast exposure as the main cause of combat casualties, comprising 55% of combat injuries during this period [4].

The impact of TBI arises from the mechanical stress caused by a primary injury, followed by progressive damage of the brain parenchyma through secondary injury mechanisms. Multiple biochemical cascades are triggered by brain damage, resulting in reactive oxygen species (ROS) production alongside blood loss and hypoxia [5]. A number of intricate pathophysiological processes, including the release of cytokines, activation of chemoreceptors, neuroinflammation, and cell injury and death, can be triggered in TBI. It also causes oxidative stress and various cerebrovascular dysfunctions, which often result in a substantial impairment of cerebral blood flow autoregulation [6]. In a blast, the explosion can induce TBI by a direct pressure wave to the skull or through excessive pressure in the vascular system [7,8,9]. These mechanisms potentially induce the brain to enter a phase of rapid acceleration–deceleration and rotation, often seen in whiplash, resulting in widespread axonal damage and neuronal death. In particular, blast TBI has devastating effects on the brain’s vasculature and triggers intra-cranial hypertension and edema. In the aftermath of a blast exposure, brain damage symptoms are characterized by cerebrovascular injury, inflammation, neuronal death, and synaptic loss. Depending on the site and severity of brain injury, TBI can cause long-lasting cognitive behavioural symptoms such as memory deficits, executive function impairment, confusion, decrease in consciousness, dizziness, concentration difficulties, slurred speech, and emotional disturbance, as well as compromised motor functions and neurological symptoms, such as epilepsy [10,11,12]. TBI can also lead to long-term complications, which may encompass a reduction in life expectancy, neurotrauma, seizure disorders, psychiatric disorders, and the onset of neurodegenerative diseases [13,14,15,16].

Since the primary injury can only be managed through prevention, treatment for TBI has until recently been focused on the alleviation of secondary injuries by targeting the mechanisms of oxidative damage either by inhibiting lipid peroxidation or through the enzymatic scavenging of superoxide radicals [17,18]. Antioxidants are ideal therapeutic agents to mitigate TBI pathologies due to their biocompatibility and effectiveness in scavenging reactive oxygen species (ROS) in the prevention of oxidative damage [19]. However, current therapies based on the above strategy have failed to show satisfactory efficacy in clinical trials due to their reduced blood–brain barrier (BBB) permeability, hydrophobicity, short half-life, and poor bioavailability in the brain [20]. On the other hand, hydrophilic carbon clusters may be used as therapeutic high-capacity antioxidants [21].

Our labs developed a biologically compatible class of oxidized carbon nanoparticles, poly-ethylene-glycol-functionalized hydrophilic carbon clusters (PEG-HCCs) [22], as prospective neuroprotective agents for TBI [23]. Also, functional and structural improvement is observed when treated with a catalytic carbon nano-antioxidant (PEG-HCC) in experimental TBI complicated by hypotension and resuscitation [24]. These particles were initially thought to act solely as high-capacity catalytic superoxide dismutase mimetics [25] but more recently were discovered to have more general enzymatic activities, including the ability to span the electron transfer complexes in mitochondria [26] and oxidize hydrogen sulphide to protective polysulphides [27], all actions that would favour neuroprotection, demonstrated in both in vitro and in vivo models. Furthermore, PEG itself has the ability to restore the integrity of cell membranes [28] but accounts for less than 10% of the superoxide actions of the PEG-HCCs [25]. Moreover, carbon particles improve cerebrovascular dysfunction post-TBI given their antioxidant nature [29]. These multiple catalytic features are consistent with PEG-HCCs acting as redox mediators, also termed nanozymes [26].

Based on an in vitro study, PEG-HCCs efficiently reduce intracellular oxidative stress and improve brain endothelial cell viability [29]. Moreover, antioxidant carbon particles are known to improve cerebrovascular dysfunction following TBI [29]. In an acute TBI model, PEG-HCCs were notably effective in TBI complicated by hypotension, a common co-morbid condition associated with head trauma [24].

In summary, the biologically compatible catalytic superoxide dismutase mimetic carbon-based nanoclusters, PEG-HCCs, can exert a promising role in preventing oxidative stress-mediated cell death by detoxifying ROS (such as SO and OH radicals) [25]. In addition, PEG-HCC treatment decreased hydrogen peroxide levels after the reperfusion of a transient middle cerebral artery occlusion (tMCAO) in hyperglycemic rats [30]. On the other hand, the PEG-HCC scavenges the OH and SO radicals when T lymphocytes specifically internalize the PEG-HCCs. These mechanisms decrease T cell-mediated inflammation in an MS (multiple sclerosis) animal model [31]. When neurons are incubated with sodium cyanide (a mitochondrial complex IV inhibitor), the PEG-HCC protects neuronal cells from hydrogen peroxide toxicity [26]. Additionally, PEG-HCCs are capable of selectively transforming harmful superoxide radicals to dioxygen and hydrogen peroxide faster than the single active site enzymes, thereby playing a role in cytoprotection [25].

Keeping the aforesaid PEG-HCC properties in mind, we have used an open blast rat TBI model in a real-time battle scenario to investigate the neuroprotective efficacy of a sub-class of PEG-HCCs.

## 2. Material and Methods

### 2.1. Preparation of PEG-HCC Nanoparticles

The PEG-HCC, developed by Tour, Kent, and colleagues [21,23,29,32] and used in the present experiment, was prepared 3–5 days prior to the experiments at the Department of Chemistry, Rice University, as previously described and characterized [22]. Briefly, highly oxidized carbon clusters (HCCs) were synthesized through the oxidative treatment of single-walled carbon nanotubes (SWCNTs) using a concentrated mixture of fuming sulfuric acid and nitric acid. This produces carbon-based materials with a variety of oxygen-rich functional groups. To improve solubility in water and saline, 4000 MW amino-methoxy poly(ethylene glycol) (mPEG_5000-NH2) was covalently attached to the HCCs via carbodiimide coupling to form PEG-functionalized HCCs (PEG-HCC). The PEG-HCC was transferred to the experimental site in Singapore by courier post immediately after its preparation. Prior work indicates that these particles are stable at ambient temperatures.

### 2.2. Animals

Thirty-four male Sprague Dawley rats (NUS Laboratory Animal Centre, National University Singapore), weighing 320–350 g each and at 3 months of age, were used for the study. A total of 28 animals were exposed to an open blast (16 PEG-HCC and 12 SALINE group animals) and 6 animals, not exposed to the blast and not exposed to treatment, served as controls (CONTROL group). Sixteen rats were intraperitoneally (IP) injected with PEG-HCC dissolved in saline (PEG group, dosage: see below), whereas the other 12 animals were injected with pure saline as a vehicle control (SALINE group). Half of the animals in each group (PEG with eight animals and SALINE with six animals) were sacrificed on day 3 after the blast, whereas the other half of the animals were sacrificed on day 14 for the collection of brains.

All handling and care of animals in this study adhered to the guidelines stipulated by the Institutional Animal Care and Use Committees (IACUC) of the Defence Science Organisation National Laboratories (project approval number: DSO/IACUC/13-141) and of the Singapore Experimental Medical Centre (project approval number: 2013/SHS/0862). Measures were taken to minimize the number of rats used and their suffering, as per the principles of the 3Rs.

### 2.3. Blast Exposure

**Figure 1:**
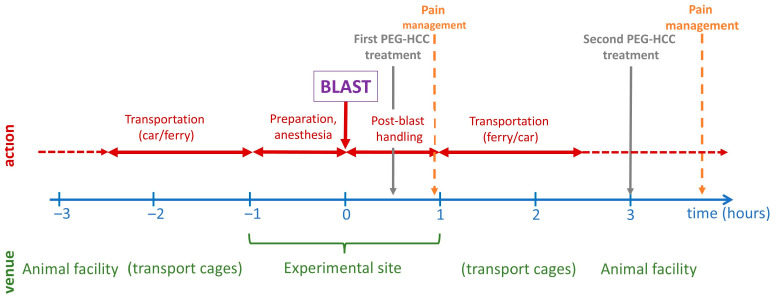
The timeline of the acute experiment from 3 h pre−blast to 3 h post−blast, showing transportation, blast exposure, and injection of the animals.

The blast experiments were performed in an experimental site dedicated to open blast explosions, as previously described [5,13]. As previously shown, this blast would cause significant non-lethal brain damage. The animals were transported from their housing facility to the site and back via land and sea transport; the temporal sequence of the events on the experimental day are shown in Figure 1. The blast was carried out offshore in an isolated experimental military site. A total of 120 kg of 2,4,6-trinitrotoluene (TNT) with a penta-erythritol tetra-nitrate (PETN) booster was detonated at a height of 1 m. Three metal cages were set up at a distance of 15–17 m and at a height of 1, 2, and 3 m(s), respectively, from the blast source with a pressure transducer adjacent to each cage. In the blasts, the peak blast overpressure values in the cages were between 13.0 psi (89.6 kPa, 0.88 atm) and 75.2 psi (518.5 kPa, 5.12 atm) (average: 23.9 ± 16.2 psi or 164.8 ± 111.7 kPa, 1.63 ± 1.10 atm), and the impulse ranged between 46.3 psi/msec and 116.9 psi/msec (average: 68.3 ± 19.0 psi/msec). Anesthesia was induced at the blast site prior to fixation in the blast cages.

Under continuing anesthesia (Ketamine (150 mg/kg) and Xylazine (10 mg/kg) intraperitoneally), rats were subjected to explosive blast overpressure after being secured to the custom-made wire caging with Velcro. The anesthetized animals were placed in such a way that their head was facing the direction of the blast pressure. Body armour was placed on the animals to protect them from the shock waves. The ears of the animals were taped down with surgical micropore tape to prevent direct blast pressure to the tympanic membrane. In addition, aqueous gel was spread on the whiskers, eye and face region, and any parts of the body which were exposed to the air to prevent any instances of burn injury. When all the animals were in position, the blast protocol proceeded. Following the blast exposure, the animals were immediately removed from the cage and assessed for any gross or penetrating facial and body injuries. The animals were returned to their transport cages approximately 60 min after the blast exposure prior to transportation back to the animal facility. Animals were allowed to recover from anesthesia and the physiological conditions of animals regarding any weight loss, distress, pain, or suffering were monitored continuously after injury.

### 2.4. Treatment Protocol and Immediate Post-Blast Management

Animals received the first dose of the PEG-HCC treatment (2 mg/kg body weight) 30–40 min after the blast (i.e., within the “golden hour” of human trauma [33,34]) at the experimental site and received the second dose (2 mg/kg body weight) 150 min after the first dose (i.e., 3 h after the blast) in their “home environment” (the animal house). Sterile physiological saline solution or drug (2 mg/kg of body weight) was injected in 0.5 mL volume IP. While intravenous (IV) injection provides a more rapidly available therapy, IP injection was selected given the difficulties in maintaining IV in the field; thus, this may be more realistic should a mass casualty event occur. Note that IP PEG-HCC injection has a slow uptake that peaks around 36 h and in vivo PEG-HCC has a circulating half-life of 27 h [31]. Dosing was established in in vitro cellular protection studies and then translated to in vivo dosing based on rat volume of distribution and intravenous administration. Efficacy was found in in vivo models of mild trauma and ischemia/reperfusion utilizing the doses comparable to the intraperitoneal doses used here [31]. Carprofen (4 mg/kg) was provided subcutaneously for pain management during drug treatment at 60 min post-injury prior to transportation back to the animal facility and subsequently upon return to the animal facility (Figure 1). All animals survived the blast and transport.

### 2.5. Chronic Post Blast Management

After animal recovery, the rats were monitored for their physical activities. The animals were allowed free access to food and water. For the first 2 days post-blast, the individually numbered animals in the SALINE and PEG groups were monitored closely for their behaviour and food intake. The animals were regularly weighed and recorded for their weight loss/gain. No animals showed any sign of severe illness or stress that required further treatment or euthanasia. On the third day, half of the animals were sacrificed, and their brains were harvested. On the fourteenth day, the remaining animals were sacrificed, and their brains were harvested.

### 2.6. Immunohistochemistry

**Figure 2:**
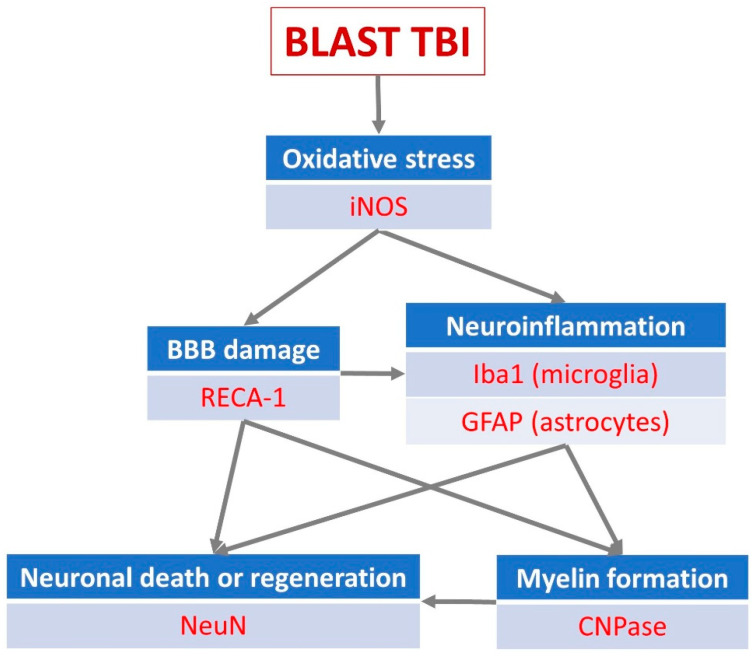
The post-traumatic events in brain parenchyma, their interrelationships, and the neuronal markers used for their visualization and quantification in the present study. BBB—blood–brain barrier; CNPase—2′,3′-cyclic-nucleotide 3′-phosphodiesterase; GFAP—glial fibrillary acidic protein; Iba1—ionized calcium-binding adaptor molecule 1; iNOS—inducible nitric oxide synthase; NeuN—neuronal nuclei; RECA-1—rat endothelial cell antigen 1.

For immunohistochemistry (IHC) exploration of the effects of the open blast explosion and the therapeutic effects of PEG-HCC on brain tissue, six antibodies were selected with a dilution of 1:1000, focusing on various biochemical cellular outcome measures critically important for the interpretation of the efficacy of PEG-HCC on the post-blast tissue damage. These were (1) neuronal nuclei (NeuN) (provider: Abcam, Cambridge, UK; host: rabbit), measuring neuronal death [35,36]; (2) inducible nitric oxide synthase (iNOS) (provider: Abcam; host: rabbit), measuring nitric oxide (NO) production via nitric oxide synthase (NOS) up- or downregulation [37]; (3) 2′,3′-cyclic-nucleotide 3′-phosphodiesterase (CNPase) (provider: BioLegend, San Diego CA, USA; host: mouse), measuring myelin integrity [38]; (4) ionized calcium-binding adaptor molecule 1 (Iba1) (provider: Abcam; host: goat), an indicator of microglia activation [39]; (5) glial fibrillary acidic protein (GFAP) (provider: Genetex, Irvine CA, USA; host: rabbit), measuring levels of neuroinflammation [40]; and (6) rat endothelial cell antigen 1 (RECA-1) (provider: Thermo Fisher, Lenexa KS, USA; host: mouse), a cell surface antigen which is expressed by all rat endothelial cells and indicates endothelial loss or reconstruction [41,42]. The selection of primary antibodies followed the selection of the outcome measures which we aimed to explore and interpret in detail, whereby the selected outcome measures represent landmark events in the pathophysiological processes following a TBI impact on brain tissue and potential sites for modulation by PEG-HCC based on the properties summarized in the Introduction (Figure 2).

From the euthanized animals on day 3, the brains of eight (8) “PEG animals” and six (6) “SALINE animals” were used for IHC studies and, similarly, from the animals euthanized on day 14, eight (8) “PEG animals” and six (6) “SALINE animals” were used for IHC studies. The six CONTROL animals were euthanized under identical conditions and their brains were harvested in an identical manner. The statistical analysis is based on the number of animals used in this study.

Brains were preserved by flash freezing. Frozen brains were sectioned at 14 μm thickness and subjected to a tris-buffered saline Triton (TBST) wash. For immunofluorescence experiments, sections were incubated with 3% bovine serum albumin (BSA) for 30 min to block nonspecific binding before incubation with the primary antibody (NeuN, RECA-1, iNOS, CNPase, Iba1, and GFAP, respectively) overnight at 4 °C. Slides were washed with TBST before incubation with species-specific fluorescent secondary antibodies for 1 h at room temperature, washed with TBST, counterstained with 4′,6-diamidino-2-phenylindole (DAPI), and mounted with Vectashield anti-fade mounting medium. For the experiments, cortical samples were taken from the frontal, parietal, and temporal lobes; however, in the case of NeuN IHC, samples were also taken from the hippocampus and the dentate gyrus.

### 2.7. Image Analysis, Statistical Analysis, and Data Presentation

Slides were imaged with the ZEISS LSM-800 microscope (ZEISS, Oberkochen, Germany), and fluorescent positive cells were quantified with Image-J software (version 1.53p; NIH). The statistical analysis is based on the above number of animals. Statistical significance was evaluated by a two-way ANOVA test in Excel (Microsoft). The data showed a normal distribution. All data are presented as mean ± SD. A *p*-value of less than 0.05 (two-tailed) was considered statistically significant for all comparisons. During the image analysis procedure, the area of labelled cells or compartments within the whole field of view (1 × 1 mm square) was measured and expressed as a proportion of the total number of cells within the whole field. For each experiment, the value obtained in the control conditions was regarded as 100%, whereas all other values were normalized to it. Thus, in the figures, the averaged data are expressed in relative terms (in %) (the coloured columns) and the relevant SD values are indicated by “empty boxes” on top of the coloured columns.

## 3. Results

### 3.1. Quantification of Neuronal Loss

**Figure 3:**
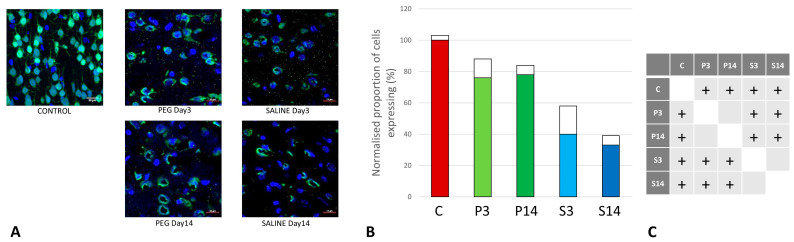
(**A**) Cortex nuclei immunofluorescence analysis of neurons stained with antibodies to neuronal nuclei (NeuN) (green) and counterstained with 4′,6-diamidino-2-phenylindole (DAPI) for nuclei (blue): examples from the CONTROL (C), PEG (P), and SALINE (S) groups analyzed 3 (P3, S3) and 14 (P14, S14) days post-blast. (**B**) Average levels of NeuN (normalized to the control) in the PEG and SALINE groups. Coloured bar = mean; open bar = 1 S.D. (**C**) Statistically significant differences (+ refers to *p* < 0.05).

**Figure 4:**
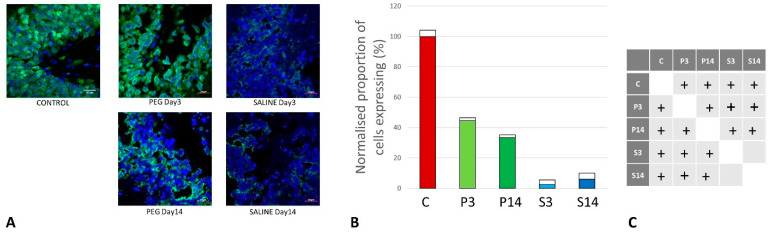
(**A**) Dentate gyrus nuclear immunofluorescence analysis, labelling neurons with antibodies to neuronal nuclei (NeuN) (green) and counterstained with 4′,6-diamidino-2-phenylindole (DAPI) for nuclei (blue): examples from the CONTROL (C), PEG (P), and SALINE (S) groups analyzed 3 (P3, S3) and 14 (P14, S14) days post-blast. (**B**) Average levels of NeuN (normalized to the control) in the PEG and SALINE groups. Coloured bar = mean; open bar = 1 S.D. (**C**) Statistically significant differences (+ refers to *p* < 0.05).

**Figure 5:**
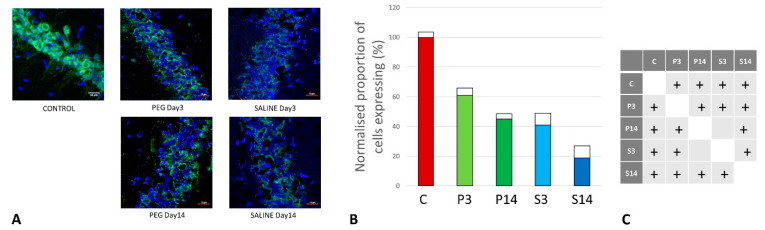
(**A**) Hippocampus nuclear immunofluorescence analysis labelling neurons with antibodies to neuronal nuclei (NeuN) (green) antibodies and counterstained with 4′,6-diamidino-2-phenylindole (DAPI) for nuclei (blue): examples from the CONTROL (C), PEG (P), and SALINE (S) groups analyzed 3 (P3, S3) and 14 (P14, S14) days post-blast. (**B**) Average levels of NeuN (normalized to the control) in the PEG and SALINE groups. Coloured bar = mean; open bar = 1 S.D. (**C**) Statistically significant differences (+ refers to *p* < 0.05).

As cell survival, cell loss, and cell regeneration are key biomarkers of the post-traumatic mechanisms in brain parenchyma, NeuN (an indicator of neuronal population) was used to visualize the loss of neurons following the blast impact. NeuN is a soluble nuclear protein, which can serve as a neuronal marker by binding to the DNA in post-mitotic neurons of the vertebrate nervous system [43,44]. In the cortex and hippocampal regions (CA1 and dentate gyrus) a significant neuronal loss was observed in the vehicle control (SALINE-treated) compared to the PEG-HCC groups, which indicates the protection of neurons by PEG-HCC-treated animals. The brain regions such as the cortex (Figure 3), dentate gyrus (Figure 4), and hippocampus (Figure 5) showed significant neuroprotection by PEG-HCC compared to SALINE groups.

However, a significant reduction in neurons even with the PEG-HCC was found compared to the control, which clearly depicts damage caused by the blast. The protective impact of PEG-HCCs in the cortex lasted up to 14 days, while in the dentate gyrus and hippocampus, the protective effect was reduced to 14 days but remained significantly higher than with saline, indicating that the neuroprotection continued and sustained the injured cells.

### 3.2. Quantification of BBB Damage

**Figure 6:**
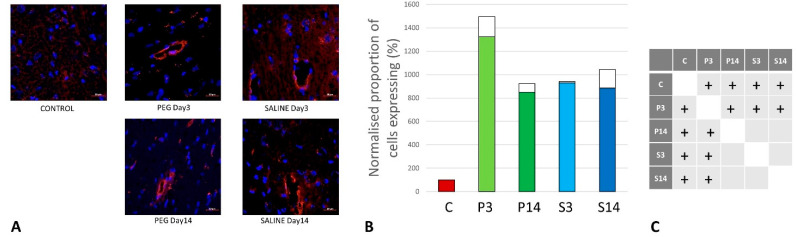
(**A**) Rat endothelial cell antigen (RECA-1) (red) immunofluorescence labelling of cortical neurons that are counterstained with 4′,6-diamidino-2-phenylindole (DAPI) for nuclei (blue): examples from the CONTROL (C), PEG (P), and SALINE (S) groups analyzed 3 (P3, S3) and 14 (P14, S14) days post-blast. (**B**) Average levels of RECA-1 (normalized to the control) in the PEG and SALINE groups. Coloured bar = mean; open bar = 1 S.D. (**C**) Statistically significant differences (+ refers to *p* < 0.05).

On day 3, RECA-1 increased significantly with PEG-HCC compared to SALINE, indicating a higher endothelial activity to repair the BBB damage. At day 14, the endothelial activity had fallen to the same level as in SALINE, indicating a transitory stimulation of endothelial activity by PEG-HCCs (Figure 6).

### 3.3. Quantification of Inflammation

**Figure 7:**
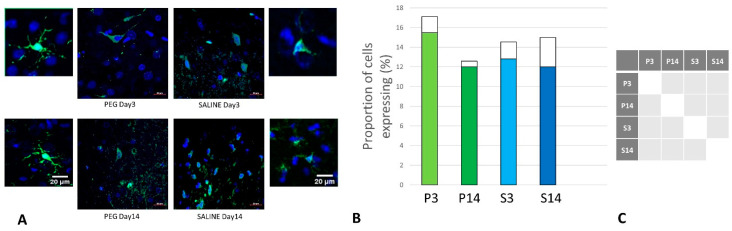
(**A**) Immunofluorescence labelling of neurons that are stained with antibodies to ionized calcium-binding adaptor molecule 1 (Iba1) (green) and counterstained with 4′,6-diamidino-2-phenylindole (DAPI) (blue) for nuclei in the cortex: examples from the PEG (P) and SALINE (S) groups analyzed 3 (P3, S3) and 14 (P14, S14) days post-blast. The smaller frames are higher-magnification views showing representative microglia cells. (**B**) The average number of labelled cells/field of view in the PEG and SALINE groups. Coloured bar = mean; open bar = 1 S.D. (**C**) Statistically significant differences (+ refers to *p* < 0.05).

Iba1 is a neuroinflammation marker expressed in microglia and an indicator of microglia activation [45,46]. The control group data are missing in this case due to technical reasons; nonetheless, no significant difference in the number of the Iba1+ microglia was observed across the PEG and SALINE groups (Figure 7).

**Figure 8:**
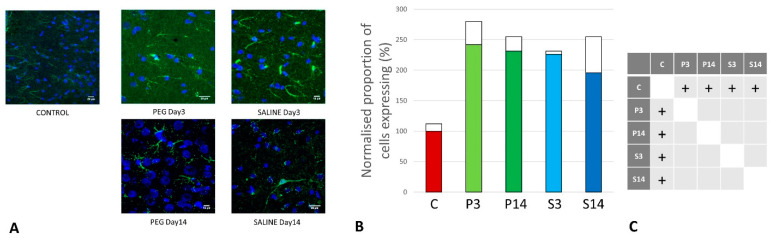
(**A**) Immunofluorescence labelling of neurons that are stained with antibodies to glial fibrillary acidic protein (GFAP) (green) and counterstained with 4′,6-diamidino-2-phenylindole (DAPI) (blue) for nuclei in the cortex: examples from the CONTROL (C), PEG (P), and SALINE (S) groups analyzed 3 (P3, S3) and 14 (P14, S14) days post-blast. (**B**) The average levels of GFAP (normalized to the control) in the PEG and SALINE groups. Coloured bar = mean; open bar = 1 S.D. (**C**) Statistically significant differences (+ refers to *p* < 0.05).

However, whereas the well-ramified versions of microglial cells were the dominant cell forms in the PEG groups (3 days and 14 days), the ameboid forms of microglial cells were the dominant cell forms in the SALINE groups. GFAP is a neuroinflammatory marker expressed in astrocytes and is an indicator of astrocytic activation [40]. GFAP activity significantly increased in the PEG and SALINE groups compared to the control (Figure 8).

Visually, hypertrophy and ramifications were observed in the SALINE group as compared to PEG, showing substantial astrogliosis. On the 14th day, PEG-HCC-treated brains clearly showed the slow recovery/repair process of astrocytes. Brightly stained artefacts found in the slices could possibly indicate glial scar formation. Although there is no significant difference in Iba1 or GFAP between the PEG-HCC and SALINE groups, there was a clear morphological difference, which indicates that PEG-HCCs have a protective effect even after 14 days.

### 3.4. Quantification of iNOS-Positive Glial Cells

Inducible nitric oxide synthase (iNOS) is an inflammatory biomarker expressed in pro-inflammatory M1 microglia. Following TBI, iNOS is expressed in response to inflammatory stimuli and is a major producer of nitric oxide (NO). NO can react with superoxide to form peroxynitrite, a powerful toxic oxidant involved in secondary tissue injury. Thus, it is an indicator of inflammation and oxidative damage after TBI [47,48].

**Figure 9:**
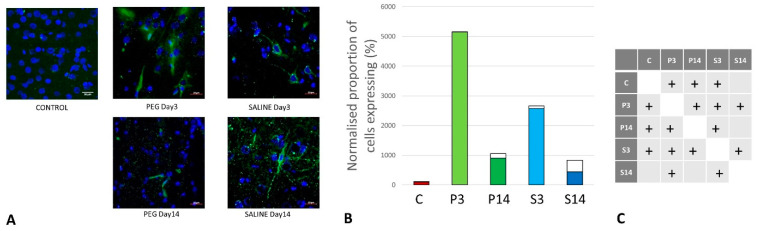
(**A**) Immunofluorescence labelling of neurons that are stained with antibodies to inducible nitic oxide synthase (iNOS) (green) and counterstained with 4′,6-diamidino-2-phenylindole (DAPI) (blue) for nuclei in the cortex: examples from the CONTROL (C), PEG (P), and SALINE (S) groups analyzed 3 (P3, S3) and 14 (P14, S14) days post-blast. (**B**) The average levels of iNOS (normalized to the control) in the PEG and SALINE groups. Coloured bar = mean; open bar = 1 S.D. (**C**) Statistically significant differences (+ refers to *p* < 0.05).

Figure 9 depicts the anti-iNOS IF labelling of brain sections from rats treated with PEG or saline following injury. iNOS was clearly induced in both PEG and SALINE groups, although the induction of iNOS was significantly lower in the SALINE group at day 3 compared to the PEG group. At day 14, the levels of iNOS were greatly reduced, with no significant difference between PEG and SALINE, although the PEG levels were still significantly raised compared to CONTROL. The lower level of induction of iNOS in the SALINE group suggests a partial impairment of the inflammatory response that is alleviated in the PEG group.

### 3.5. Quantification of Oligodendrocyte Regeneration

**Figure 10:**
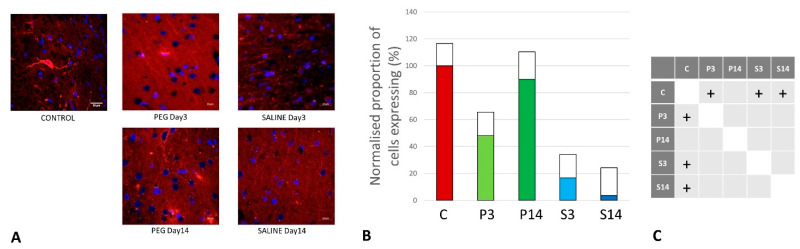
(**A**) Immunofluorescence labelling of neurons that are stained with antibodies to 2′,3′-cyclic-nucleotide 3′-phosphodiesterase (CNPase) (red) and counterstained with 4′,6-diamidino-2-phenylindole (DAPI) (blue) for nuclei in the cortex: examples from the CONTROL (C), PEG (P), and SALINE (S) groups analyzed 3 (P3, S3) and 14 (P14, S14) days post-blast. (**B**) Average levels of CNPase (normalized to the control) in the PEG and SALINE groups. Coloured bar = mean; open bar = 1 S.D. (**C**) Statistically significant differences (+ refers to *p* < 0.05).

CNPase is expressed in oligodendrocytes and is an indicator of myelin integrity. An increase in CNPase is an indicator of oligodendrocyte regeneration [49,50]. CNPase was significantly reduced at day 3 but returned to normal levels at day 14 with PEG-HCCs, whereas they remained at very low levels with saline (Figure 10). Although the differences between P14 and P3, S3, or S14 were not significant, these observations are an indication of the protection of myelin regeneration by PEG-HCCs.

## 4. Discussion

We report here an open blast TBI model that offers a clinically realistic injury and treatment paradigm [5]. Histological markers of injury demonstrated evidence of multiple parameters of injury, some mitigated significantly by PEG-HCC treatment.

The blast model used in this study was chosen to give principal focus on the direct effects on the brain. With the rat lying prone with the head fixed facing the blast in an open field, the dominant effect would be a primary blast injury on the brain with minimal higher-order blast injuries from penetration, acceleration/deceleration, or blood loss. The blast would result in a transient energy wave, caused by the short-lived blast impulse, travelling through the brain in a rostral-to-dorsal direction and affecting all parts of the brain. With an open field, there would be minimal reflections amplifying or focusing the blast force. Since the rest of the animal bodies were protected by body armour, there would be minimal indirect effects on the brain through the respiratory or circulatory systems [51,52]. At the site of blast exposure, the level of the blast force the rats were exposed to (blast overpressure 89.6–518.5 kPa, mean 164.8 kPa) was non-lethal. In humans, this level of blast force would cause concussion and long-term effects such as PTSD [53,54].

In a previous study using a similar blast model, animals that survived the explosion exhibited noticeable alterations in brain structure, metabolism, and inflammation, indicating potential damage to the brain. The persistence of certain structural changes even in later stages is concerning, suggesting these changes could be long-lasting, resulting in significant functional impairments [5]. Standard clinical imaging tools typically lack the sensitivity to detect ultrastructural changes in the brain resulting from mild blast traumatic brain injury. Therefore, the absence of obvious changes in routine clinical assessments does not guarantee the absence of injuries. In our previous study, histopathological examinations revealed ultrastructural changes in animals that were not detectable by standard clinical imaging tools [5].

Our previous findings also demonstrate a dose–response relationship between blast overpressure and observed changes, especially in large animals (i.e., non-human primates). For instance, animals exposed to higher levels of blast showed more pronounced behavioural deficits, scalp hematomas (no subarachnoid hemorrhage or subdural hemorrhage was observed), and histopathological changes compared to those exposed to lower levels of blast [13].

To date, there are no FDA-approved therapies for the treatment of traumatic brain injury. Here, we studied the pathological consequences of blast TBI and showed how PEG-HCCs protect neurons against TBI-caused damage by using immunostainings of injury-related markers. Our findings from a rat open blast TBI model provided evidence for the neuroprotective effects of PEG-HCCs when administered within the golden hour after the blast, even with IP dosing that may not provide the drug immediately to the brain. We infer that the acute dosage of PEG-HCCs as used in this study provided a significant protective effect but not complete protection, with some of the effects enduring for the 14-day experimental period.

Neurons undergo necrosis during primary injury and continue to undergo apoptosis due to the continuous effects of secondary injury [55]. Blast-induced TBI can be due to a direct blast wave to the skull or overpressure from the vascular system due to disruptions in BBB integrity following blast exposure [7]. The direct blast wave may whiplash the brain in a violent motion inside the skull, resulting in diffuse axonal damage that eventually causes neuronal death. Higher NeuN immunoreactivity in the PEG-HCC groups indicates neuroprotection after treatment, potentially by the inhibition of neuroinflammation activated by necrotic cell bodies, thus preventing inflammation-activated apoptosis. Additionally, we observed increased NeuN immunoreactivity in the cortex region at 14 days than at 3 days post-treatment, which is a probable sign of healing. However, after 14 days, the dentate gyrus and CA1 of the hippocampus showed a decline in NeuN immunoreactivity, suggesting that the effects of PEG-HCCs may only be transient at these sites.

Neuronal damage greatly increases iNOS expression, with peak concentrations reaching 1–2 days after TBI. In response to the cytokines, iNOS is predominantly expressed in glial cells. During inflammation, NO is continually produced by iNOS until the synthase is degraded [56]. By inhibiting ferroptosis (iron-dependent cellular death), iNOS keeps the M1 microglia cells alive. Consequently, TBI increases neuroinflammation, whereas treatment with L-NIL (iNOS inhibitor) alleviates the inflammatory process and elevates the expression of ferroptosis proteins [57].

In this study, we saw a significantly higher induction of iNOS in the PEG-HCC animals compared to the SALINE animals at day 3, indicating that the TBI stimulated this inducible protein. However, an increase in NO by itself is not nearly as toxic as it is when combined with the superoxide radical, as that yields the very toxic peroxynitrate and protein nitrosylation. Moreover, the oxidation of nitric oxide synthase inhibits dimerization, leading to uncoupling and the generation of superoxide rather than NO [58]. We have previously shown that PEG-HCCs do not quench the NO radical [25] and that PEG-HCCs restore the balance between NO and SO following trauma [29]. Given that NO is required for protective actions such as anti-inflammatory and vasodilation, while excess levels have some innate toxicity, we consider overall a net benefit to have quenched the superoxide radical.

We used Iba1 and GFAP antibodies to detect the activated microglia and reactive astrocytes in order to examine the activation of this inflammation pathway. Though there was no discernible difference between the PEG-HCC and SALINE groups in terms of numbers of glial cells, we observed changes in microglia morphology. As seen in Figure 7A, microglia exist in its ramified resting state in the PEG-HCC-treated group and in an activated amoeboid state in the SALINE group. In their typical ramified shape, microglia constantly survey for debris and foreign bodies. Microglia become activated and convert to their phagocytic amoeboid state in response to the severe injury. These amoeboid microglia are responsible for the release of pro-inflammatory cytokines [59,60]. When compared to PEG-HCCs, the GFAP-positive reactive astrocytes showed some cellular hypertrophy and a higher degree of ramification in the SALINE groups, indicating an elevated astrogliosis. Acute CNS injury causes astrocyte morphological alteration and changes in molecular expression, as well as the formation of glial scars. If the triggering mechanism has been resolved, there is a possibility for resolution and neuroprotection in mild astrogliosis, resulting in no overlap of reactive astrocytes and glial scars [61]. Our observations on the state of the neuroinflammatory cells are consistent with a recently published blast overpressure experiment comparing single and repeated blasts, in which a change in morphology for microglia and GFAP for animals exposed to a single blast and an increase in immunoreactivity in the frontal cortex for those exposed to repeated blasts [62].

As part of post-blast pain management, the animals were treated with Carprofen, for both PEG-HCC and SALINE animals equally. Carprofen is a non-steroidal anti-inflammatory drug that inhibits cyclooxygenase and could therefore partially contribute to some of the anti-inflammatory effects in the brain. However, since both PEG-HCC and SALINE animals were treated with Carprofen, the potential contribution of Carprofen can be ignored. Furthermore, Carprofen was only administered in direct connection with the acute experiment and would not be expected to still have any effect at day 3 or 14 after the blast. The difference between PEG-HCC and SALINE with regard to the inflammatory markers Iba1 and GFAP was qualitative rather than quantitative, indicating a mitigating effect of PEG-HCCs.

CNPase was chosen as a myelin-producing oligodendrocyte marker because it is abundantly expressed in oligodendrocytes and Schwann cells [63]. The myelin marker CNPase is related to oligodendrocyte maturation and is widely expressed in pre-myelinating and myelinating oligodendrocytes [64,65]. We observed an increased level of CNPase expression in PEG-HCC groups, which may be related to oligodendrocyte regeneration. Oxidative stress from TBI can cause the oligodendrocytes to undergo apoptosis. Because of extremely poor glutathione production, oligodendrocytes are very susceptible to damage caused by oxidative stress [66]. Apoptosis can also be induced by pro-inflammatory cytokines released by the amoeboid reactive microglia. At day 14, the SALINE group animals showed a drop in CNPase expression. This may highlight an endogenous mechanism that replaces the oligodendrocyte population through the proliferation of oligodendrocyte precursor cells. A few groups reported an increase in the number of Olig2+ cells after injury [67,68]. Our findings indicate that between days 3 and 14 of post-TBI, the PEG-treated groups showed an increase in oligodendrocytes, returning to levels similar to CONTROL. It is apparent that the PEG-HCC accelerated the oligodendrogenesis process. We also note the higher CNPase expression in PEG-HCC-treated groups 3 days post-TBI when compared to the saline-treated group at the same time point, although it was not significantly different. This led us to infer that the PEG-HCC plays a clear role in protecting the oligodendrocytes from undergoing apoptosis, possibly through its antioxidant properties discussed above.

## 5. Conclusions

The open blast rat model used focuses on direct blast effects and injuries of the brain. The blast would result in a short standing energy wave that progressed with very high energy through the whole brain, resulting in direct lesions and spalling and shearing effects. Blast loads create very brief acceleration durations that may cause distinct neurophysiological outcomes. We used our existing patented PEG-HCCs with antioxidant properties to treat oxidative stress caused by TBI [22]. Previous studies have indicated that PEG-HCCs can recover and maintain the brain’s structural and homeostatic environment by reducing neuronal and glial cell death. In this study, we found PEG-HCCs to significantly ameliorate neuronal loss in the brain cortex, the dentate gyrus, and hippocampus and to improve blood–brain barrier integrity. The different states of the activation of microglia and astrocytes seen across time in the various cohorts of the study indicate the intricate and complex roles of PEG-HCCs in the management of neuroinflammation and neuronal and glial death. Our current experimental data support the efficacy of our carbon clusters, PEG-HCCs, against post-TBI effects and indicate a potential therapeutic role of PEG-HCCs in the treatment of blast-induced TBI.

## Data Availability

Data unavailable.

## Abbreviations

BBB—blood-brain-barrier; CA1—CA1 layer of the hippocampus; CNPase—2′,3′-cyclic-nucleotide 3′-phosphodiesterase; DAPI—4′,6-diamidino-2-phenylindole; GFAP—glial fibrillary acidic protein; Iba1—ionized calcium-binding adaptor molecule 1; IHC—immunohistochemistry; iNOS—inducible nitric oxide synthase; IP—intraperitoneal; NeuN—neuronal nuclei; PEG-HCCs—poly-ethylene-glycol-functionalized hydrophilic carbon clusters; RECA-1—rat endothelial cell antigen 1; ROS—reactive oxygen species; TBI—traumatic brain injury; TBST—tris-buffered saline with 0.1% Tween^®^ 20 detergent.

## References

[1] Maas A.I.R., Menon D.K., Adelson P.D., Andelic N., Bell M.J., Belli A., Bragge P., Brazinova A., Büki A., Chesnut R.M. (2017). Traumatic brain injury: Integrated approaches to improve prevention, clinical care, and research. Lancet Neurol..

[2] Daugherty J., Waltzman D., Sarmiento K., Xu L. (2019). Traumatic brain injury–related deaths by race/ethnicity, sex, intent, and mechanism of injury—United States, 2000–2017. Morb. Mortal. Wkly. Rep..

[3] DePalma R.G., Hoffman S.W. (2018). Combat blast related traumatic brain injury (TBI): Decade of recognition; promise of progress. Behav. Brain Res..

[4] Bukowski J., Nowadly C.D., Schauer S.G., Koyfman A., Long B. (2023). High risk and low prevalence diseases: Blast injuries. Am. J. Emerg. Med..

[5] Pun P.B.L., Kan E.M., Salim A., Li Z., Ng K.C., Moochhala S.M., Ling E.A., HongTan M., Lu J. (2011). Low level primary blast injury in rodent brain. Front. Neurol..

[6] Gardner A.J., Zafonte R. (2016). Neuroepidemiology of traumatic brain injury. Handb. Clin. Neurol..

[7] Kabu S., Jaffer H., Petro M., Dudzinski D., Stewart D., Courtney A., Coutney M., Labhasetwar V. (2015). Blast-Associated Shock Waves Result in Increased Brain Vascular Leakage and Elevated ROS Levels in a Rat Model of Traumatic Brain Injury. PLoS ONE.

[8] Meabon J.S., Huber B.R., Cross D.J., Richards T.L., Minoshima S., Pagulayan K.F., Li G., Meeker K.D., Kraemer B.C., Petrie E.C. (2016). Repetitive blast exposure in mice and combat veterans causes persistent cerebellar dysfunction. Sci. Transl. Med..

[9] Phipps H., Mondello S., Wilson A., Dittmer T., Rohde N.N., Schroeder P.J., Nichols J., McGirt C., Hoffman J., Tanksley K. (2020). Characteristics and Impact of U.S. Military Blast-Related Mild Traumatic Brain Injury: A Systematic Review. Front. Neurol..

[10] Georges A., Das J.M. (2024). Traumatic brain injury (Archive). StatPearls [Internet].

[11] Girgis F., Pace J., Sweet J., Miller J.P. (2016). Hippocampal neurophysiologic changes after mild traumatic brain injury and potential neuromodulation treatment approaches. Front. Syst. Neurosci..

[12] Schimmel S.J., Acosta S., Lozano D. (2017). Neuroinflammation in traumatic brain injury: A chronic response to an acute injury. Brain Circ..

[13] Lu J., Ng K.C., Ling G., Wu J., Poon D.J.F., Kan E.M., Tan M.H., Wu Y.J., Li P., Moochhala S. (2012). Effect of blast exposure on the brain structure and cognition in *Macaca fascicularis*. J. Neurotrauma.

[14] Bramlett H.M., Dietrich W.D. (2015). Long-Term Consequences of Traumatic Brain Injury: Current Status of Potential Mechanisms of Injury and Neurological Outcomes. J. Neurotrauma.

[15] Gardner R.C., Burke J.F., Nettiksimmons J., Golgman S., Tanner C.M., Yaffe K. (2015). Traumatic brain injury in later life increases risk for Parkinson disease. Ann. Neurol..

[16] Graham N.S., Sharp D.J. (2019). Understanding neurodegeneration after traumatic brain injury: From mechanisms to clinical trials in dementia. J. Neurol. Neurosurg. Psychiatry.

[17] Lu J., Moochhala S., Shirhan M., Ng K.C., Teo A.L., Tan M.H., Moore X.L., Wong M.C., Ling E.A. (2003). Neuroprotection by aminoguanidine after lateral fluid-percussive brain injury in rats: A combined magnetic resonance imaging, histopathologic and functional study. Neuropharmacology.

[18] Di Pietro V., Yakoub K.M., Caruso G., Lazzarino G., Signoretti S., Barbey A.K., Tavazzi B., Lazzarino G., Belli A., Amorini A.M. (2020). Antioxidant Therapies in Traumatic Brain Injury. Antioxidants.

[19] Kumar H., Bhardwaj K., Nepovimova E., Kuca K., Dhanjal D.S., Bhardwaj S., Bhatia S.K., Verma R., Kumar D. (2020). Antioxidant functionalized nanoparticles: A combat against oxidative stress. Nanomaterials.

[20] Forman H.J., Zhang H. (2021). Targeting oxidative stress in disease: Promise and limitations of antioxidant therapy. Nat. Rev. Drug Discov..

[21] Samuel E.L.G., Duong M.T., Bitner B.R., Marcano D.C., Tour J.M., Kent T.A. (2014). Hydrophilic carbon clusters as therapeutic, high-capacity antioxidants. Trends Biotechnol..

[22] Tour J.M., Berlin J., Marcano D., Leonard A., Kent T.A., Pautler R.G., Bitner B., Inoue T. (2017). Use of Carbon Nanomaterials with Antioxidant Properties to Treat Oxidative Stress. U.S. Patent.

[23] Marcano D.C., Bitner B.R., Berlin J.M., Jarjour J., Lee J.M., Jacob A., Fabian R.H., Kent T.A., Tour J.M. (2013). Design of poly (ethylene glycol)-functionalized hydrophilic carbon clusters for targeted therapy of cerebrovascular dysfunction in mild traumatic brain injury. J. Neurotrauma.

[24] Mendoza K., Derry P.J., Cherian L.M., Garcia R., Nilewski L., Goodman J.C., Mbye L., Robertson C.S., Tour J.M., Kent T.A. (2019). Functional and Structural Improvement with a Catalytic Carbon Nano-Antioxidant in Experimental Traumatic Brain Injury Complicated by Hypotension and Resuscitation. J. Neurotrauma.

[25] Samuel E.L.G., Marcano D.C., Berka V., Bitner B.R., Wu G., Potter A., Fabian R.H., Pautler R.G., Kent T.A., Tsai A.-L. (2015). Highly efficient conversion of superoxide to oxygen using hydrophilic carbon clusters. Proc. Natl. Acad. Sci. USA.

[26] Derry P.J., Nilewski L.G., Sikkema W.K.A., Mendoza K., Jalilov A., Berka V., McHugh E.A., Tsai A.-L., Tour J.M., Kent T.A. (2019). Catalytic oxidation and reduction reactions of hydrophilic carbon clusters with NADH and cytochrome C: Features of an electron transport nanozyme. Nanoscale.

[27] Derry P.J., Liopo A.V., Mouli K., McHugh E.A., Vo A.T.T., McKelvey A., Suva L.J., Wu G., Gao Y., Olson K.R. (2024). Oxidation of Hydrogen Sulfide to Polysulfide and Thiosulfate by a Carbon Nanozyme: Therapeutic Implications with an Emphasis on Down Syndrome. Adv. Mater..

[28] Shi R. (2013). Polyethylene glycol repairs membrane damage and enhances functional recovery: A tissue engineering approach to spinal cord injury. Neurosci. Bull..

[29] Bitner B.R., Marcano D.C., Berlin J.M., Fabian R.H., Cherian L., Culver J.C., Dickinson M.E., Robertson C.S., Pautler R.G., Kent T.A. (2012). Antioxidant Carbon Particles Improve Cerebrovascular Dysfunction Following Traumatic Brain Injury. ACS Nano.

[30] Fabian R.H., Derry P.J., Rea H.C., Dalmeida W.V., Nilewski L.G., Sikkema W.K.A., Mandava P., Tsai A.-L., Mendoza K., Berka V. (2018). Efficacy of Novel Carbon Nanoparticle Antioxidant Therapy in a Severe Model of Reversible Middle Cerebral Artery Stroke in Acutely Hyperglycemic Rats. Front. Neurol..

[31] Huq R., Samuel E.L.G., Sikkema W.K.A., Nilewski L.G., Lee T., Tanner M.R., Khan F.S., Porter P.C., Tajhya R.B., Patel R.S. (2016). Preferential uptake of antioxidant carbon nanoparticles by T lymphocytes for immunomodulation. Sci. Rep..

[32] Sahni D., Jea A., Mata J.A., Marcano D.C., Sivaganesan A., Berlin J.M., Tatsui C.E., Sun Z., Luerssen T.G., Meng S. (2013). Biocompatibility of pristine graphene for neuronal interface. J. Neurosurg. Pediatr..

[33] Lerner E.B., Moscati R.M. (2001). The golden hour: Scientific fact or medical “urban legend”?. Acad. Emerg. Med..

[34] Clarke J.R., Trooskin S.Z., Doshi P.J., Greenwald L., Mode C.J. (2002). Time to laparotomy for intra-abdominal bleeding from trauma does affect survival for delays up to 90 minutes. J. Trauma.

[35] Gundersen H.J., Jensen E.B. (1987). The efficiency of systematic sampling in stereology and its prediction. J. Microsc..

[36] West M.J., Slomianka L., Gundersen H.J. (1991). Unbiased stereological estimation of the total number of neurons in the subdivisions of the rat hippocampus using the optical fractionator. Anat. Rec..

[37] Kaur C., Singh J., Moochhala S., Lim M.K., Lu J., Ling E.A. (1999). Induction of NADPH diaphorase/nitric oxide synthase in the spinal cord motor neurons of rats following a single and multiple non-penetrative blasts. Histol. Histopathol..

[38] Gravel M., Peterson J., Yong V.W., Kottis V., Trapp B., Braun P.E. (1996). Overexpression of 2′,3′-cyclic nucleotide 3′-phosphodiesterase in transgenic mice alters oligodendrocyte development and produces aberrant myelination. Mol. Cell. Neurosci..

[39] Zheng R., Lee K., Qi Z., Wang Z., Xu Z., Wu X., Mao Y. (2022). Neuroinflammation Following Traumatic Brain Injury: Take It Seriously or Not. Front. Immunol..

[40] Middeldorp J., Hol E.M. (2011). GFAP in health and disease. Prog. Neurobiol..

[41] Eng L.F., Ghirnikar R.S., Lee Y.L. (2000). Glial fibrillary acidic protein: GFAP-thirty-one years (1969–2000). Neurochem. Res..

[42] Cattin A.L., Burden J.J., Van Emmenis L., Mackenzie F.E., Hoving J.J., Garcia Calavia N., Guo Y., McLaughlin M., Rosenberg L.H., Quereda V. (2015). Macrophage-induced blood vessels guide Schwann cell-mediated regeneration of peripheral nerves. Cell.

[43] Mullen R.J., Buck C.R., Smith A.M. (1992). NeuN, a neuronal specific nuclear protein in vertebrates. Development.

[44] Wolf H.K., Buslei R., Schmidt-Kastner R., Schmidt-Kastner P.K., Pietsch T., Wiestler O.D., Blümcke I. (1996). NeuN: A useful neuronal marker for diagnostic histopathology. J. Histochem. Cytochem..

[45] Ito D., Imai Y., Ohsawa K., Nakajima K., Fukuuchi Y., Kohsaka S. (1998). Microglia-specific localisation of a novel calcium binding protein, Iba1. Brain Res. Mol. Brain Res..

[46] Ohsawa K., Imai Y., Sasaki Y., Kohsaka S. (2004). Microglia/macrophage-specific protein Iba1 binds to fimbrin and enhances its actin-bundling activity. J. Neurochem..

[47] Nathan C., Xie Q.W. (1994). Nitric oxide synthases: Roles, tolls, and controls. Cell.

[48] Colton C.A., Gilbert D.L. (1987). Production of superoxide anions by a CNS macrophage, the microglia. FEBS Lett..

[49] Baumann N., Pham-Dinh D. (2001). Biology of oligodendrocyte and myelin in the mammalian central nervous system. Physiol. Rev..

[50] Dyer C.A., Benjamins J.A. (1989). Organization of oligodendroglial membrane sheets. I. Association of myelin basic protein and 2′,3′-cyclic nucleotide 3′-phosphohydrolase with cytoskeleton. J. Neurosci. Res..

[51] Bryden D.W., Tilgham J.I., Hinds S.R. (2019). Blast-related traumatic brain injury: Current concepts and research considerations. J. Exp. Neurosci..

[52] Cernak I., Kobeissy F.H. (2015). Blast Injuries and Blast-Induced Neurotrauna: Overview of Pathophysiology and Experimental Kwoledge Models and Findings. Brain Neurotrauma: Molecular, Neuropsychological, and Rehabilitation Aspects.

[53] Rosenfeld J.V., McFarlane A.C., Bragge P., Armonda R.A., Grimes J.B., Ling G.S. (2013). Blast-related traumatic brain injury. Lancet Neurol..

[54] Champion H.R., Holcomb J.B., Young L.A. (2009). Injuries from explosions: Physics, biophysics, pathology, and required research focus. J. Trauma.

[55] Royo N.C., Schouten J.W., Fulp C.T., Shimizu S., Marklund N., Graham D.I., McIntosh D.K. (2003). From cell death to neuronal regeneration: Building a new brain after traumatic brain injury. J. Neuropathol. Exp. Neurol..

[56] Garry P.S., Ezra M., Rowland M.J., Westbrook J., Parrinson K.T.S. (2015). The role of the nitric oxide pathway in brain injury and its treatment--from bench to bedside. Exp. Neurol..

[57] Qu W., Cheng Y., Peng W., Wu Y., Rui T., Luo C., Zhang J. (2022). Targeting iNOS Alleviates Early Brain Injury After Experimental Subarachnoid Hemorrhage via Promoting Ferroptosis of M1 Microglia and Reducing Neuroinflammation. Mol. Neurobiol..

[58] Fabian R.H., Kent T.A. (2012). Hyperglycemia accentuates persistent “functional uncoupling” of cerebral microvascular nitric oxide and superoxide following focal ischemia/reperfusion in rats. Transl. Stroke Res..

[59] Kettenmann H., Hanisch U.K., Noda M., Verkhratsky A. (2011). Physiology of microglia. Physiol. Rev..

[60] Stoll G., Jander S. (1999). The role of microglia and macrophages in the pathophysiology of the CNS. Prog. Neurobiol..

[61] Sofroniew M.V. (2009). Molecular dissection of reactive astrogliosis and glial scar formation. Trends Neurosci..

[62] Svetlov S.I., Prima V., Glushakova O., Svetlov A., Kirk D.R., Gutierrez H., Serebruany V.L., Curley K.C., Wang K.K.W., Hayes R.L. (2012). Neuro-glial and systemic mechanisms of pathological responses in rat models of primary blast overpressure compared to “composite” blast. Front. Neurol..

[63] Sprinkle T.J., Agee J.F., Tippins R.B., Chamberlain C.R., Faguet G.B., DeVries G.H. (1987). Monoclonal antibody production to human and bovine 2′,3′-cyclic nucleotide 3′-phosphodiesterase (CNPase): High-specificity recognition in whole brain acetone powders and conservation of sequence CNP1 and CNP2. Brain Res..

[64] Trapp B.D., Bernier L., Andrews S.B., Colman D.R. (1988). Cellular and subcellular distribution of 2′,3′-cyclic nucleotide 3′-phosphodiesterase and its mRNA in the rat central nervous system. J. Neurochem..

[65] Verrier J.D., Jackson T.C., Gillespie D.G., Janesko-Feldman K., Bansal R., Goebbels S., Nave K.-A., Kochanek P.M., Jackson E.K. (2013). Role of CNPase in the oligodendrocytic extracellular 2′,3′-cAMP-adenosine pathway. Glia.

[66] French H.M., Reid M., Mamontov P., Simmons R.A., Grinspan J.B. (2009). Oxidative stress disrupts oligodendrocyte maturation. J. Neurosci. Res..

[67] Dent K.A., Christie K.J., Bye N., Basrai H.S., Turbic A., Habgood M., Cate H.S., Turnley A.M. (2015). Oligodendrocyte birth and death following traumatic brain injury in adult mice. PLoS ONE.

[68] Flygt J., Clausen F., Marklund N. (2017). Diffuse traumatic brain injury in the mouse induces a transient proliferation of oligodendrocyte progenitor cells in injured white matter tracts. Restor. Neurol. Neurosci..

